# Cancer care in rural areas

**DOI:** 10.1038/sj.bjc.6601769

**Published:** 2004-03-23

**Authors:** R Colomer, J R Germà, R Rosell, J M Borràs

**Affiliations:** 1 Institut Català d'Oncologia, Hospital Dr Josep Trueta Girona, 17007, Girona, Spain; 2 Institut Català d'Oncologia, l'Hospitalet, Barcelona; 3 Institut Català d'Oncologia, Badalona, Barcelona, Spain

Sir,

Stevenson *et al* (2003) have analysed the level of consensus on the priorities for cancer care among health professionals who treat rural cancer patients in Northeast Scotland. Cancer care in this health area centralises oncology specialists in the city of Aberdeen, although approximately half of its 500 000 population live in rural areas. The authors found a single disagreement among the participant health professionals: which is the best location for chemotherapy delivery (central *vs*. local). In our opinion, such a predicament should be formulated in a different way, so that one alternative does not rule out the other. The question should possibly be whether local treatment can be compatible with central treatment in a public health system.

The organizational structure of the Catalan Institute of Oncology (ICO) has addressed the issue of decentralization of cancer care and treatment delivery, and may provide valuable experience to help formulating a more appropriate question. The ICO is the major cancer care provider in Catalonia, serving a population of 2.5 million in three cancer centers: Duran i Reynals (l'Hospitalet, Barcelona), Dr Josep Trueta (Girona), and Germans Trias i Pujol (Badalona, Barcelona). The ICO began its operations in 1995 in l'Hospitalet, and expanded to Girona and Badalona in 2002 and 2003, respectively. A major strength of the organization has been its ability to reach agreements between each of the three centres and the community hospitals in their respective regions. The agreements are flexible, and allow for oncology specialists consultation only and for full chemotherapy prescription and administration. The health network established by ICO allows for the planning of chemotherapy delivery according to community hospital needs and also by cancer type, the chemotherapy regimen, and the complexity of treatment. For example, some community hospitals deliver most types of chemotherapy to the majority of cancer patients, while others deliver only adjuvant chemotherapy in breast or colon cancer patients. Patients included in clinical trials are usually treated centrally during the study protocol. The agreements are signed annually and can be modified.

Our cancer care experience strongly points out that the future of cancer treatment delivery in rural areas must not be either local or central, but rather a judicious combination of the two.

## References

[bib1] Stevenson L, Campbell NC, Kiehlmann PA (2003) Providing cancer services to remote and rural areas: consensus study. Br J Cancer 89: 821–827 12942111 10.1038/sj.bjc.6601166 PMC2394474

